# Primary tonsillar tuberculosis: a forgotten clinical identity

**Published:** 2019-12

**Authors:** Seyyed Jafar Motahari, Parvaneh Afshar, Maryam Ghasemi, Lale Vahedi Larijani, Somayeh Sheidaei

**Affiliations:** 1 Clinical Research Development Unit of Bou-Ali Sina Hospital, Mazandaran University of Medical Sciences, Sari, Iran; 2 Research and Development of Referral Laboratory, Deputy of Health Management, Mazandaran University of Medical Sciences, Sari, Iran; 3 Department of Pathology, School of Medicine, Mazandaran University of Medical Sciences, Sari, Iran; 4 Gastrointestinal Cancer Research Center, Mazandaran University of Medical Sciences, Sari, Iran

**Keywords:** Tonsil, Tuberculosis, *Mycobacterium tuberculosis*, Lymphadenopathy

## Abstract

Primary tonsillar tuberculosis is an uncommon entity and a diagnostic challenge. Misdiagnosis can be prevented with early professional para-clinical finding. The true diagnosis is often delayed and infection management depends on recognizing disease patterns and early laboratory documentation.

This rare clinical caseation granuloma with positive clinical symptoms, negative results of radiology/laboratory and alone based on histopathological finding without any *Mycobacterium* particle indicates the role of an accurate laboratory/pathology finding for urgent medical intervention treatment and lifesaving of patients, particularly in immunocompromised group.

## INTRODUCTION

Tuberculosis (TB) is a bacterial contagious chronic infectious disease caused by *Mycobacterium tuberculosis*. *Mycobacterium tuberculosis* is an obligate aerobe, motionless, non-spore-forming, acid-fast bacillus (1). The disease infects a wide variety of mammalian species. TB occurs more frequently among male relative to female, particularly in productivity age adults. TB is an airborne disease that typically presented with pulmonary involvement but can also appear in extrapulmonary tuberculosis (EPTB) form in other organs of the human body (about 15% patients) such as, the lymph nodes, the urinary tract and the abdomen, bones, brain, the kidneys, spine and even the skin (2). However, the low number of TB bacilli in the tissues of many infected animals meaning that it frequently led to false-negative results (3). Tuberculosis remains a leading cause of mortality and morbidity in all the world especially in developing and developed countries today due to immunodeficiency diseases (as HIV infection, diabetes, chronic liver or kidney disease, cancers), poor nutrition, inadequate living conditions and multidrug-resistant (MDR) tuberculosis (4).

Tuberculosis infection generally involves the lungs. The tongue and palate are very common form, but localization in tonsillar is a rare (5). Tuberculosis of the oral cavity which is an uncommon occurrence can be primary or secondary (5). According to this point, TB rarely involves the tonsils, as the antiseptic effect and cleansing action of saliva, the presence of saprophytes in the oral cavity, and a thick layer of squamous cells protect them. The secondary form of tonsillar tuberculosis is more common than primary (5). Primary tonsillar TB accounts for less than 0.5% of TB cases and is, therefore, a diagnostic challenge.

The aim of this report is to point out that primary tuberculosis lesion in tonsils including the presentation and differential diagnoses. Due to its rarity, primary tonsillar TB is difficult to diagnose dental extraction, low immunity, and direct exposure may be predisposing factors.

## CASE DESCRIPTION

A 18-year-old woman presented with a persistent sore throat, painful swallowing, and fever, loss of weight and appetite, and enlargement of the lymph nodes of the neck in Northeast of Iran, was admitted to the Bou-Ali Sina Hospital of Mazandaran University of Medical Sciences, Sari, Iran. The symptoms started two months prior to the emergence of the lesion, a physical examination revealed cervical lymphadenopathy in the anterior right and left the chain of the neck and enlargement of the bilateral area. She has used antibiotic orally cefalexin 500 mg/ BID during 2 weeks with the impression of bacterial infection, then ten days after completed the treatment she was given metronidazole and cefazolin (IV), but did not respond to them and treatment was failed.

A chest wall examination was normal. She had no previous history suggestive of tuberculosis or any other major illness in the family, and no prior history of anti-tubercular therapy. She was not a smoker and alcohol consumption. Table 1 shows the Para-clinical finding testes (Radiology and laboratory) except pathology asses test. The results were normal.

**Table 1.** The para-clinical findings except pathology asses test

**Section**	**Test**	**Result**
Radiology	Chest X-ray	Normal
	Lung CT scan	Normal
Laboratory	Hemoglobin	9.4 g/dL, Normal
	HCT	31.4%, Normal
	MCV	59.4 fL, Normal
	MCH	17.8 Pg, Normal
	ESR	26 mm, Normal
	PT, PTT, INR	Normal
	CRP	Negative, Normal
	CMV IgM	Negative, Normal
	Toxo IgM	Negative, Normal
	HIV (Ag and Ab)	Negative, Normal
	Liver and Renal function tests	Normal
	Mantoux test (skin test)	Scar equal to 10 mm, Normal
	Three consecutive sputum assay based on Ziehl–Neelsen staining of direct smear and culture in Lowenstein–Jensen medium for acid-fast bacilli	Negative, Normal
	Paraffin-embedded block PCR assay by DNA-based nucleic acid amplification for *Mycobacterium Tuberculosis*	Negative, Normal

## RESULTS

**Fig. 1.**
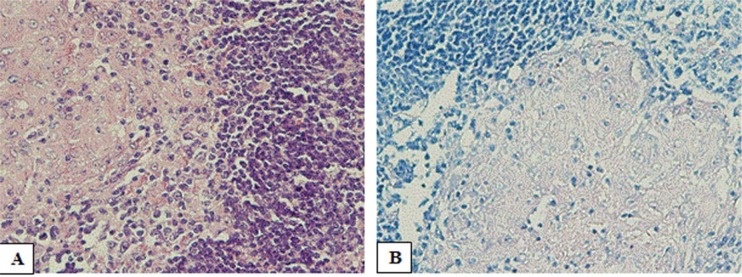
(A) Microphotograph of histopathology of resected specimen of tonsils showing caseous granuloma including germinal centers, epithelioid granulomas with Langerhans cell histiocytosis and foreign body giant cells, together with necrosis areas. (PAS stain, ×10); (B) Ziehl-Neelsen staining for acid-fast bacilli is negative.

Thirty-seven HIV-positive while only 1 (4 percent) healthy individual had the same pattern (Fig. 1). These results show a statistically significant difference between the two groups (P-value = 0.007). The rest of the comparison the two groups were calculated (Table 1).

A tonsillectomy performed to rule out Sarcoidosis, Wegener’s granulomatosis, and malignancy. Histopathology sections study showed a granulomatous lesion, with epithelioid giant cells, small central caseous necrosis, and Langerhans-type giant cells. However, PAS, Ziehl–Neelsen and modified Ziehl–Neelsen staining for fungal element, acid-fast bacilli, and Nocardia organism were negative, respectively (Fig. 1).

Based on the histopathological findings, the patient was given first line anti TB drugs including isoniazid, rifampicin, pyrazinamide, and ethambutol). After two weeks of the treatment, the patient’s signs and symptoms regressed and were completely resolved. Treatment was continued for 6 months with rifampicin and isoniazid).

## DISCUSSION

Extrapulmonary TB is responsible for 25% of cases of TB-related morbidity (6). TB of the tonsils is rare and may be either primary or secondary. Primary tuberculosis was seen as an uncommon of initial infection form and usually occur in the younger patient, while secondary tuberculosis is more common and is seen mostly in older persons, particularly when health status declines (5, 7). The tonsils may become infected via contact with material and sputum samples containing *Mycobacterium tuberculosis* (6). In the past, prevalence tonsillar tuberculosis was relatively high due to *Mycobacterium bovis* infection through the ingestion of unpasteurized cow milk (8). From the pathological viewpoint, epithelioid granulomas with caseous necrosis, and Langhans giant cells or foreign body giant cells (FBGC) with or without acid-fast bacilli are typical features of tonsillar TB (9). In EPTB should be performed Chest X-ray, lung CT scan and sputum smear for acid-fast bacilli due exclude pulmonary involvement. Due to its rarity, primary tonsillar TB is difficult to diagnose, and TB direct exposure, tooth extraction, inadequate dental hygiene, periodontitis, leukoplakia, low immunity, human immunodeficiency virus (HIV) infection and resistance to drug treatment are considered predisposing factors for tonsillar tuberculosis (8, 10). The common signs and symptoms of primary tonsillar TB are a sore throat, enlargement of the tonsils, and cervical lymphadenopathy (11). Other diseases, such as hematological disorders, actinomycosis, syphilis, Wegener’s disease, and malignancy, have a similar presentation and must be ruled out too (6).

On the other hand, absence of the Mantoux test reactivity in TB infected individuals can occur in immunocompromised persons, or persons newly infected with TB, or in persons with miliary TB (12). Thus, early diagnosis and treatment is necessary to reduce mortality. In addition to other specific laboratory tests are mandatory for the identification of tubercle bacilli a histopathological examination with Ziehl Neelson stain should be performed for the definite diagnosis (7). Even if all confirmatory TB tests are negative, Epithelioid granulomas with caseous necrosis, Langhans’ and foreign body giant cells with/without acid fast bacilli are typical features of tonsillar TB (13). Hence the excellent pathological is required for the Tonsillar tuberculosis identification.
